# Complete Genome Sequence of Rhodococcus ruber R1, a Novel Strain Showing a Broad Catabolic Potential toward Lignin-Derived Aromatics

**DOI:** 10.1128/MRA.00905-19

**Published:** 2020-01-09

**Authors:** Carlos Farkas, Raúl A. Donoso, Felipe Melis-Arcos, Carla Gárate-Castro, Danilo Pérez-Pantoja

**Affiliations:** a Programa Institucional de Fomento a la Investigación, Desarrollo, e Innovación, Universidad Tecnológica Metropolitana, Santiago, Chile; b Center of Applied Ecology and Sustainability (CAPES), Santiago, Chile; University of Southern California

## Abstract

Rhodococcus ruber R1 was isolated from a pulp mill wastewater treatment plant because of its ability to use methoxylated aromatics as growth substrates. We report the 5.56-Mb genome sequence of strain R1, which can provide insights into the biodegradation of lignin-derived phenolic monomers and potentially support processes for lignocellulose conversion.

## ANNOUNCEMENT

**FIG 1:**
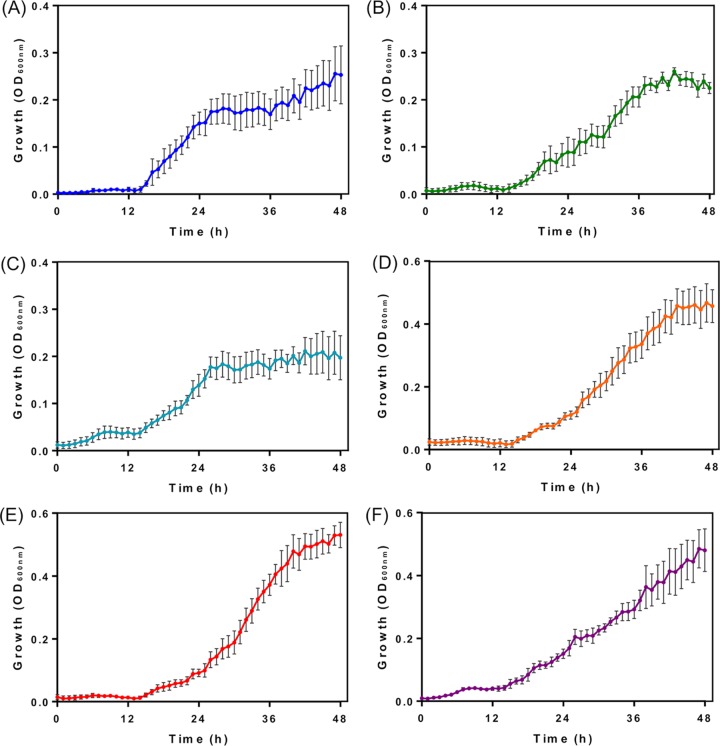
Growth of Rhodococcus ruber R1 on several lignocellulose-related aromatic compounds. The growth of strain R1 on 2 mM guaiacol (A), *p*-cresol (B), *p*-coumarate (C), phenol (D), *m*-cresol (E), and 4-hydroxybenzoate (F) as the sole carbon and energy sources was assessed. All reagents were of analytical grade and were obtained from Sigma-Aldrich (Buchs, Switzerland). The cultures were incubated in a 96-well microplate (Thermo Fisher Scientific, Rochester, NY, USA), with agitation, for 15 min at 30°C, and the optical density at 600 nm (OD_600_) was measured with a Synergy HTX multimode reader (BioTek, Winooski, VT, USA). Minimal salt medium without carbon sources (control) supported minor growth, which was subtracted from the growth curves for medium with carbon sources. Cell cultures were inoculated with 100-fold dilutions of overnight cultures of strain R1 grown on R2A broth. Five replicates were performed for growth measurements. Error bars represent 1 standard deviation.

Members of the *Rhodococcus* genus are aerobic, GC-rich, Gram-positive bacteria belonging to the actinomycetes (1), whose recognized capacity to degrade hydrocarbons suggests that they might be useful in lignin biodegradation (2–5). Here, we report the complete genome sequence of Rhodococcus ruber R1, an actinobacterium with the ability to grow on several phenolics as the sole carbon source (Fig. 1). The R1 strain was isolated using a liquid enrichment culture strategy, starting from a sludge that was collected at a pulp mill effluent treatment plant in the Biobio River basin in Chile. The cultures were first incubated at 30°C, with shaking at 120 rpm, in minimal salt medium containing 2.0 mM guaiacol as the sole carbon source. After three consecutive transfers to fresh medium, the final cultures were plated on R2A solidified medium (1% agar), and isolated colonies of strain R1 were obtained after 2 days. The strain was preliminarily identified as Rhodococcus ruber based on a partial sequence of the 16S rRNA gene obtained by sequencing of the PCR product obtained with primers 27F (5′-AGAGTTTGATCCTGGCTCAG-3′) and 1492R (5′-TACGGCTACCTTGTTACGACTT-3′) (6).

Genomic DNA of R. ruber R1 cells cultivated in R2A broth (Neogen) was obtained using the GenElute kit (Sigma-Aldrich), and sequencing was accomplished by combining Illumina and PacBio technology. Briefly, a 20-kb SMRTbell library (Pacific Biosciences) was generated, following the manufacturer’s protocol, at Macrogen, Inc. (Seoul, South Korea). Next, the PacBio RS II platform was used for single-molecule real-time (SMRT) sequencing by Macrogen, Inc., yielding 87,188 reads with an *N*_50_ value of 18,929 bases. Additionally, a genomic DNA library with a median insert size of 588 bases was prepared using the Nextera XT library kit (Illumina), following the manufacturer’s protocol, at MicrobesNG (Birmingham, UK), and the library was sequenced by using MiSeq technology (2 × 250 bp; Illumina). A total of 874,325 paired-end trimmed reads were produced from raw data using Trimmomatic v.0.39, with an average quality cutoff value of Q30 (7). Illumina and PacBio reads were assembled using Unicycler v.0.4.8 in default mode (8), yielding a final assembly of 3 circular contigs.

The genome of strain R1 consists of one circular chromosome of 5,352,622 bp (89× depth) and two plasmids of 179,109 bp (159×) and 33,016 bp (81×). A whole-genome comparative analysis was performed by calculating the average nucleotide identity based on BLAST (ANIb) against the genomes of several R. ruber strains by using JSpeciesWS v.3.0.2 (9). Strain R1 showed an ANIb value of 99% with respect to strain P14 (GenBank accession no. CP024315), indicating that it is properly designated a R. ruber strain (10). Functional annotation was performed using PGAP v.4.8 (11), identifying 4,973 coding sequences, 3 rRNA operons, and 53 tRNAs. The extensive catabolic potential of this strain is consistent with the environment from which it was isolated, since lignin decomposition during pulp production results in the formation of a broad range of monomeric phenols (12).

### Data availability.

The genome sequence of strain R1 has been deposited in GenBank under accession numbers CP038030, CP038031, and CP038032. The raw reads are associated with BioProject number PRJNA527802 and have been deposited in the SRA repository under accession numbers SRR8833178 (PacBio h5 files), SRR8833179 (PacBio subreads), and SRR10609577 (MiSeq reads).
